# Synstable Fusion: A Network-Based Algorithm for Estimating Driver Genes in Fusion Structures

**DOI:** 10.3390/molecules23082055

**Published:** 2018-08-16

**Authors:** Mingzhe Xu, Zhongmeng Zhao, Xuanping Zhang, Aiqing Gao, Shuyan Wu, Jiayin Wang

**Affiliations:** 1 Department of Computer Science and Technology, School of Electronic and Information Engineering, Xi’an Jiaotong University, Xi’an 710049, China; mingzhe.xu@hnuahe.edu.cn (M.X.); zmzhao@mail.xjtu.edu.cn (Z.Z.); algoxjtu@163.com (A.G.); 2 Department of Automation, College of Intelligent Manufacturing and Automation, Henan University of Animal Husbandry and Economy, Zhengzhou 450011, China; 3 Shaanxi Engineering Research Center of Medical and Health Big Data, School of Electronic and Information Engineering, Xi’an Jiaotong University, Xi’an 710049, China; 4 Department of Network Technology, College of Intelligent Manufacturing and Automation, Henan University of Animal Husbandry and Economy, Zhengzhou 450011, China; xxxwljys@126.com

**Keywords:** gene fusion data, gene susceptibility prioritization, evaluating driver partner, gene networks

## Abstract

Gene fusion structure is a class of common somatic mutational events in cancer genomes, which are often formed by chromosomal mutations. Identifying the driver gene(s) in a fusion structure is important for many downstream analyses and it contributes to clinical practices. Existing computational approaches have prioritized the importance of oncogenes by incorporating prior knowledge from gene networks. However, different methods sometimes suffer different weaknesses when handling gene fusion data due to multiple issues such as fusion gene representation, network integration, and the effectiveness of the evaluation algorithms. In this paper, Synstable Fusion (SYN), an algorithm for computationally evaluating the fusion genes, is proposed. This algorithm uses network-based strategy by incorporating gene networks as prior information, but estimates the driver genes according to the destructiveness hypothesis. This hypothesis balances the two popular evaluation strategies in the existing studies, thereby providing more comprehensive results. A machine learning framework is introduced to integrate multiple networks and further solve the conflicting results from different networks. In addition, a synchronous stability model is established to reduce the computational complexity of the evaluation algorithm. To evaluate the proposed algorithm, we conduct a series of experiments on both artificial and real datasets. The results demonstrate that the proposed algorithm performs well on different configurations and is robust when altering the internal parameter settings.

## 1. Introduction

Gene fusion is an important class of somatic mutational events in cancers [1]. A series of studies have shown that gene fusion structures, as well as the related genomic structural variations, are significantly associated with cancer susceptibilities across multiple cancer types [1,2,3,4,5,6]. With the development of sequencing technology, detecting gene fusion structures has become routine work in a number of computational pipelines for cancer sequencing data [7,8,9]. 

A fusion gene is typically formed by the interaction of two or more genes that are usually called partner genes. Normally, a fusion gene has a driver partner and one or more passenger partners, according to their roles in the evolution of tumor tissue [10]. The driver partner has a vital function in the carcinogenesis processes. Thus, identifying the driver partner is important for many downstream analyses and presents clinical implications. However, the throughput for validating driver genes is limited by current technology, which is both time consuming and expensive. A small number of the driver partners have demonstrated associations to cancer susceptibilities. Thus, computational approaches have been introduced to filter and prioritize the driver partner candidates, which facilitate and may further guide functional validations. To evaluate the importance of each partner in a gene fusion structure, gene networks are used in almost every existing approach, although different approaches vary in their use and application. A gene network is usually represented as a weighted graph, where each node in the graph denotes a gene, whereas each edge denotes a specific type of interaction between the two genes. Different types of approaches and interactions exist, including co-expression and co-localization networks [11,12,13], genetic interaction networks [14,15], pathway networks [16,17], physical interaction networks [18,19], shared protein domain networks [20], and predicted networks [21,22].

Along with the accumulation of gene network data, network-based approaches are faced with two major computational challenges. The first is determining how to incorporate knowledge from various types of networks. Multiple heterogeneous gene networks reflect different relationships. A common strategy involves establishing a virtual network by weighting the prior information from different networks. This is similar to the collapsing, or burden-test, strategy used in association studies [23] or the multi-source data-integration and decision-making process [24]. Benefiting from the amplification of the data signals via the newly collapsed network, the evaluation algorithms may be better for discovering potential associations and be more accurate in prioritizing the susceptibility genes [25,26,27]. Here, edge weight and graph structure are the two major evaluation strategies used to sort the important nodes (genes) through the collapsed network. The importance of edge weight is obvious, whereas the graph structure is considered by calculating the impact of each node based on the network, such as node degree [28] and node betweenness [29]. Node degree is a local topology strategy that only computes the weights on the edges that directly connect to the node. Node betweenness provides a global view by presenting the connectivity influence of nodes on the entire network. The existing approaches, however, are usually sensitive to the incorporation of the networks. When a neural network is collapsed into a disease-associated network, it may excessively dilute the data signal [23]. For example, in the multi-layer design of neural networks [24,30], multiple disease-associated network data are merged into a single output signal. Most of the data being processed within neural networks are eliminated by the weights of the input layer and the activation function of neurons in hidden layers [30].

The second major computational challenge is addressing the conflicting results from different networks. Different from the point mutation or indel calls, a gene fusion structure consists of two or more partner genes. Gene networks do not contain any “combined” nodes corresponding to a fusion gene. Thus, in many cases, the evaluation algorithms may provide conflicting results on the same virtual network. To solve the conflicts, after extensive experimental verifications [31,32,33,34], Wu et al. [34] provided the hypothesis that “if a fusion gene plays an important role in tumor formation, then the partner genes should be an important node in the gene network”. This hypothesis, called “network fusion centrality”, is based on many previous research works, which concluded that all partner genes of the carcinogenic fusion gene usually have higher network centrality, and suggested that oncogenes prefer hub nodes in the network. The network fusion centrality hypothesis allows the algorithms to merge the nodes that correspond to the partner genes into a burden node representing the fusion gene [34]. In this case, the importance of a gene fusion structure is the accumulation of the importance of the previous partner nodes. However, some of the information between the nodes, which may be lost due to the overlapped edges of merged partner gene nodes, is often ignored.

Multiple approaches are available for prioritizing partner genes, among which network fusion centrality (FC) strategy is popular [28,29,34], as it is able to process gene fusion data better than other existing approaches. In this strategy, the gene networks are obtained as prior knowledge, each of which contains a set of genes. Note that, each node of these networks represents a single gene, and each edge denotes a specific type of interaction between the two genes. As none of the nodes represent a fusion gene or a gene fusion structure, the fusion gene nodes are constructed by merging the corresponding partner genes. To achieve this, the merging step first maps the partner genes to the entire gene network, and then each partner inherits the functions of the original gene on the network to evaluate the potential influence.

Two evaluation strategies for measuring the importance of a node are widely used: node degree [28] criterion and node betweenness [29] criterion. In the node degree algorithm, the degree of node *i* is calculated with $K\left(i\right)=\frac{\sum_{j\in G}a_{ij}}{N-1}$, where *N* represents the number of nodes and *G* represents the set of nodes. For unweighted networks, $a_{ij}\in \left(0,1\right)$, where 0 indicates that no edge exists between node *i* and node *j*, and 1 indicates that an edge exists. For weighted networks, $a_{ij}$ denotes the edge weight between nodes, where *K*(*i*) represents the weighted degree of a node. The degree of the node represents the direct connection state between the node and other nodes. The importance of the node is expressed by the number of directly connected nodes. This method evaluates the significance of a node based on how well the node is directly connected to other nodes in the network topology. The advantage of this method is that the calculation is simple and the algorithm’s time complexity is $O\left(N^{2}\right)$. The disadvantage is that only the neighbors of the node are considered, and only the local importance of the nodes in the network is calculated. For nodes in different positions in a complex network, the node importance caused by various topologies is not considered.

Node betweenness is a parameter used by Freeman [29] to measure social status of individuals in their research on social networks. The betweenness of node *k* is defined as the number of shortest paths between any two nodes passing through node *k*. The betweenness centrality *B*(*k*) of node *k* is defined as $B\left(k\right)=\frac{g\left(k\right)}{g}$, where g is the number of shortest paths between each pair of nodes, and g(k) is the number of the shortest paths via node *k*. The larger the value of node betweenness, the greater the role played by the node in the connectivity between other nodes in the network. That is, the greater the influence of the node on the network connectivity, the more important the node to the entire network. The node betweenness mainly considers the impact of nodes on the connectivity between other nodes in the network. The advantage is that the global importance of a node is explained by the impact of the node on the shortest paths between nodes in the entire network. The disadvantage is that the interaction between directly connected nodes is ignored, and the method is highly complex because it is time consuming to find the shortest path between all nodes.

The algorithm based on fusion centrality degree (DEG) [28,34] uses the degree of fusion node as the evaluation measurement, whereas the algorithm based on fusion centrality betweenness (BET) [29,34] evaluates the fusion nodes based on the betweenness. However, these criteria have been further argued to have their own preferences; thus, more comprehensive strategies are suggested. Other than the degree and betweenness, graph stability is another important measurement in graph theory to describe destructiveness of a network. The graph stability state is gradually approximated if all of weights of the edges satisfy a necessary condition [35]. The necessary condition is determined by the size of the network, average connectivity among the nodes, and a coupling coefficient that relies on graph topology. Existing studies have proposed multiple synchronous stability criteria for various graph topologies [35]. For example, many networks have a semi-ring 2*K* adjacent sub-structure, which enables existing conclusions on synchronous stability criteria, widely extensible to more complicated gene network topologies. Specifically, when *k =* 1, the graph degenerates to a ring structure, whereas if *k = n/*2, the graph is a fully connected graph. Once the synchronous stability criteria are locked in the evaluation algorithm, the calculation complexity for the edge weight condition considerably decreases compared to the betweenness calculation.

To overcome the disadvantages of the current methods, and to evaluate the cancer susceptibility created by a fusion gene based on the synchronous stability method, an algorithm named Synstable Fusion is proposed in this paper. Synchronous stability means that the coupled network is synchronously stable if the internal coupling matrix and the network coupling matrix satisfy certain conditions [35]. The proposed algorithm calculates the importance of genes in the gene network according to the “destructiveness equals to importance” hypothesis [28,34,36], which states that the importance of a node in a connected graph is identical to the destructiveness of deleting the node, and evaluates the corresponding fusion genes through the importance of partner gene nodes. The Synstable Fusion algorithm, which is based on synchronous stability, evaluates the importance of the fusion gene according to the whether or not the gene network achieves a synchronously stable state. When a weighted network falls into a synchronously stable state, the network ignores the noise and insignificant information while retaining the important node edges and network structure as much as possible, thereby reducing the computational complexity when evaluating the overall impact of the node on the network. The destructiveness of deleting the node is measured by using the network difference criterion, which reflects the importance of the gene nodes. This approach not only considers the local importance of the node, but also measures the influence of the node on the overall network structure, so the gene node’s importance can be accurately calculated. The performance of our algorithm is tested and compared to the DEG and BET algorithms in a series of experiments. The experimental results demonstrate that the Synstable Fusion algorithm is able to effectively evaluate cancer fusion genes and performs better than the existing method.

## 2. Results

In order to test and verify the effectiveness of the proposed algorithm, named Synstable Fusion, we applied the algorithm to a widely used whole-gene network [36] obtained by 17 heterogeneous data to evaluate the importance of the fusion genes represented by the nodes. This gene network was obtained from Wu et al. [36], and the edge weights in the network indicate the tendency of the two genes to be joined to work together in one pathway. This network not only represents direct interactions between genes, but also includes functional interactions in a broader sense and has been used in many pathological and therapeutic studies related to cancer genes [37,38,39,40,41]. The 40,230 genes included in the entire gene network are provided in the Supplementary Materials. In order to reflect as much key and useful information as possible, the network has to be further processed to retain reliable inter-gene interactions. In the experiments, we used the “network fusion centrality” hypothesis, which was also used in many subsequent studies [42,43,44,45,46].

### 2.1. Experimental Data

The experimental data were selected based on the above studies [34,36]. A gene whose mutation is associate to a disease is called a susceptible gene. We followed the hypothesis that fusion genes formed by the interaction of susceptible cancer genes have relatively high significance, since susceptible cancer genes are important for the production of cancer [31,32,33,34]. We extracted 699 professionally curated human oncogenes from the Cancer Gene Census (CGC) [47] project as the susceptible cancer fusion genes, from which cancer may result due to their mutations. The CGC project collects and validates all published cancer-related genetic mutation studies by professionals in the field, collating them into a database with filtering criteria, and updates and maintains the data. Oncogenic mutations include both single-gene mutations (amplification, insertion, deletion, etc.) and translocations (fusions). Thus, this oncogene list also contains all possible partner genes of known oncogenic fusion genes until the date (December 2017) we obtained the list (Supplementary Table S1).

In the test data, it is assumed that $N_{f}$ represents the number of total fusion genes, $N_{i}$ is the number of susceptible fusion genes, and $N_{o}$ is non-susceptible fusion genes. So, $N_{f}=N_{i}+N_{o}$. Two partner genes form a fusion gene, thus the number of partner genes in the dataset is $2N_{f}=2N_{i}+2N_{o}$. To generate the dataset, we randomly selected $2N_{i}$ susceptible partner genes from the known susceptible cancer genes [47], then paired them to create $N_{i}$ susceptible fusion genes. For non-susceptible fusion genes, we randomly picked $2N_{o}$ common partner genes from the whole-gene network [36] and selected pairs to create $N_{o}$ ordinary fusion genes. Here, we simply used the random function in the programming language’s built-in library to implement random sampling without replacement process, and reset the random seed before each random process to ensure irregularity. $N_{i}$ important fusion genes were assembled from paired samples of 2$N_{i}$ oncogenes by random sampling without replacement. The same random sampling method was applied to 2$N_{o}$ common genes to extract pairs of genes into $N_{o}$ common fusion genes. The possibility of repeating samples inside the $N_{i}$ and $N_{o}$ datasets was avoided because the non-return sampling method was adopted. Since the whole gene network also contained 699 oncogenes for formation of susceptible fusion genes, and the selection process of $N_{i}$ and $N_{o}$ was independent of each other, overlaps between the important fusion genes ($N_{i}$) and the ordinary fusion genes ($N_{o}$) of one dataset occurred. Once this happened, we re-selected 2$N_{o}$ common genes and randomly generated $N_{o}$ fusion genes until no duplication was present between susceptible fusions and ordinary fusions. We prepared two $N_{i}$ configurations and three $N_{f}\;$($N_{f}=N_{i}+N_{o}$) configurations for the experiment, and 20 sets of random data were selected for each $N_{i}\;+\;N_{o}$ configuration, so there were 2 × 3 × 20 = 120 sets of data in total. Every set of experimental data was created accordingly. Real known oncogenic fusion genes can be created using this procedure. Three expert-curated carcinogenic fusion genes, *EWSR1-FEV*, *HMGA2-LPP*, and *EWSR1-ETV4*, were identified from the datasets. All were assessed at high importance rankings by our evaluation algorithm. The results of respective datasets are provided in Supplementary Table S2.

### 2.2. Experimental Results

The effects of the SYN algorithm are illustrated using three criteria: (1) distribution curve of susceptible fusion gene; (2) recognition rate; and (3) receiver operating characteristic curve. The test applied various values of $N_{f}$ and $N_{i}$. The effectiveness of the SYN algorithm was validated by the comparison with the DEG and BET algorithms. Different experiment scenarios were created based on various $N_{f}$ and $N_{i}$ values. For $N_{f}\in \left\{150,200,250\right\}$ and $N_{i}\in \left\{15,25\right\}$, a total of six parameter configurations were generated. For each configuration, 20 sets of data were randomly generated. Our algorithm and the other two algorithms were applied to each set to separately calculate the importance and then sort the fusion genes according to these values. The results of the different configurations and algorithms are statistically summarized and the average data calculated from the 20 results of each case are demonstrated in the following subsections.

#### 2.2.1. Distribution Curve of Susceptible Fusion Gene

**Figure 1:**
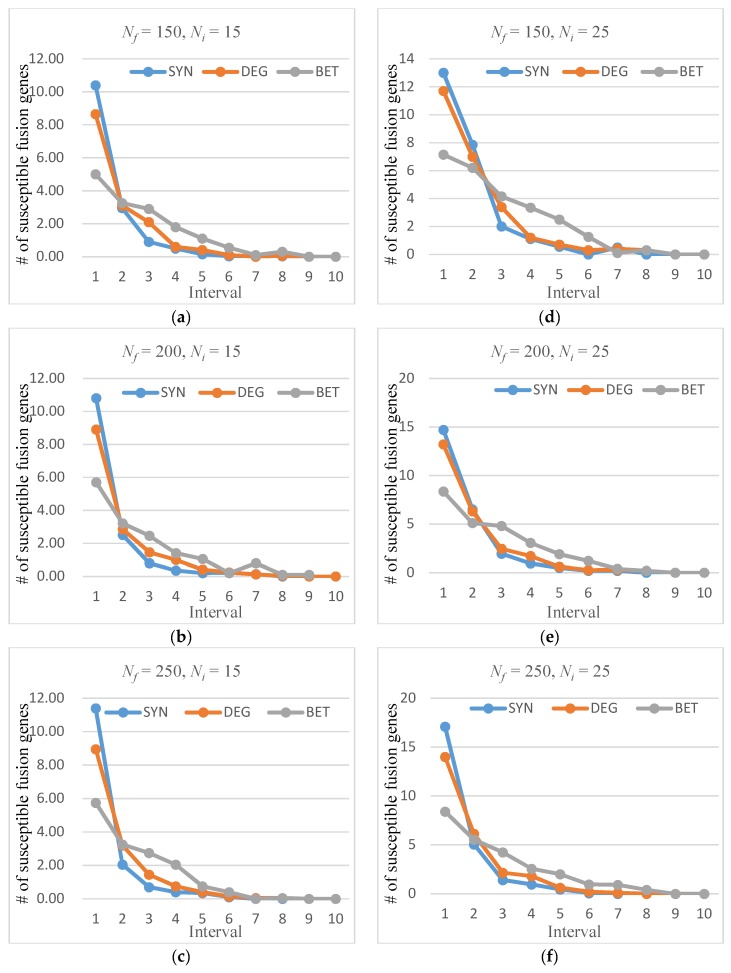
Average distribution curve of susceptible fusion genes: (**a**) $N_{f}=150$, $N_{i}=15$; (**b**) $N_{f}=200$, $N_{i}=15$; (**c**) $N_{f}=250$, $N_{i}=15$; (**d**) $N_{f}=150$, $N_{i}=25$; (**e**) $N_{f}=200$, $N_{i}=25$; and (**f**) $N_{f}=250$, $N_{i}=25$.

The susceptible fusion genes were divided into 10 intervals $I_{i}$ (*i* = 1, 2, …, 10), where $I_{i}=\left(\frac{i-1}{10}N_{f},\frac{i}{10}N_{f}\right]$. For each dataset, all calculated fusion gene significance was sorted in descending order, and then separated into 10 ranking intervals. The number of susceptible fusion genes that fell under each interval were counted. The results showed the effect of SYN, DEG, and BET algorithms in six cases of $N_{f}\in \left\{150,200,250\right\}$ and $N_{i}\in \left\{15,25\right\}$. Figure 1 shows the average distribution curves of the susceptible fusion genes identified by the three algorithms.

**Figure 2:**
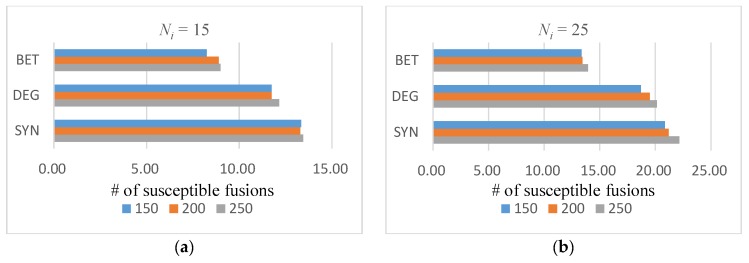
Average number of susceptible fusions in the top 20%. Different colors indicate different cases of total fusion gene amounts: (**a**) $N_{i}=15$ and (**b**) $N_{i}=25$.

From the distribution curves of the susceptible fusion genes, SYN was able to find most of the significant fusion genes from the first two intervals. In $N_{i}=15$ cases, the mean number of susceptible fusion genes in the top two intervals was 13.367, and this number was 21.4 in $N_{i}=25$ situations. There were approximately zero susceptible fusion genes in the lowest five ranges. Therefore, we summarize the average number of susceptible fusions in the top 20% ranked fusion genes in various cases in Figure 2.

From Figure 2, the average results of the SYN algorithm always outperform the results obtained with the DEG and BET methods in all situations. The largest difference occurred when $N_{i}=25$ and $N_{f}=250$: the average number of susceptible fusions found by the SYN algorithm was 58.8% more than the BET algorithm. The smallest gap occurred in comparing with the DEG algorithm when $N_{i}=15$ and $N_{f}=250$, as the difference percentage was 10.7%. From these results in Figure 2, we found that as the total number of fusion genes increased notably (by one-third or one-quarter), the amount of susceptible fusions within the top 20% area did not increase considerably, and the ratios were lower than the increasing rates of total fusion genes. We will discuss possible reasons for this result later in the Discussion section.

#### 2.2.2. Recognition Rate

**Figure 3:**
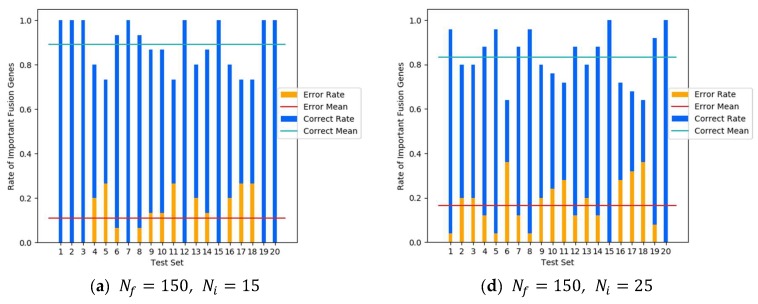

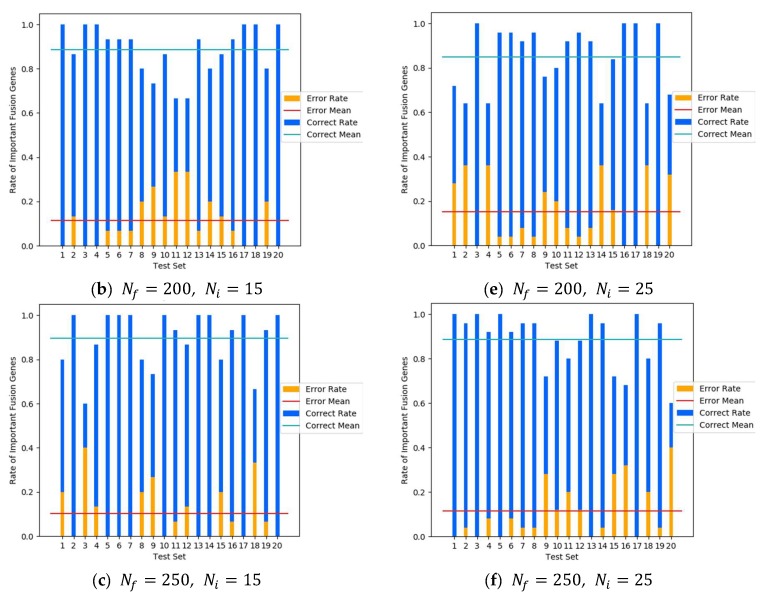
Recognition rates (correct rate) *p*(2) (blue bars) of susceptible fusion genes in the top 20% of ranked fusion genes in each experimental result. The orange bar represents the rate of important fusion genes which is not included in this interval (error rate). (**a**) $N_{f}=150$, $N_{i}=15$; (**b**) $N_{f}=200$, $N_{i}=15$; (**c**) $N_{f}=250$, $N_{i}=15$; (**d**) $N_{f}=150$, $N_{i}=25$; (**e**) $N_{f}=200$, $N_{i}=25$; and (**f**) $N_{f}=250$, $N_{i}=25$.

In order to illustrate the effectiveness of the proposed algorithm, the recognition rate of the susceptible fusion gene was adopted. The recognition rate represents the ratio of susceptible fusion genes located in a statistical interval to the total susceptible fusion genes. The recognition rate *P* is presented as $P\left(i\right)=\frac{f\left(R\left(i\right)\right)}{N_{i}}$, where R(i) denotes the *i*^th^ statistical interval, $R\left(i\right)=\left[1,\frac{i}{10}N_{f}\right]$, i∈{1,2,…,10}, and f(R(i)) indicates the number of susceptible fusion genes being found in the *i*th interval. We randomly selected 120 sets, and generated mixed experimental data in six cases ($N_{f}\in \left\{150,200,250\right\}$, $N_{i}\in \left\{15,25\right\}$). As an illustrative case, Figure 3 demonstrates the *p*(2) value of every experimental result.

The summarized average results are exhibited using radar panels, where vertices indicate the statistical intervals of the susceptible fusions, and axes indicate that recognition rate, which gradually increased outward. Because almost all susceptible fusion genes were included in the top 50% of ranked result, only the first five intervals are shown in the figures.

**Figure 4:**
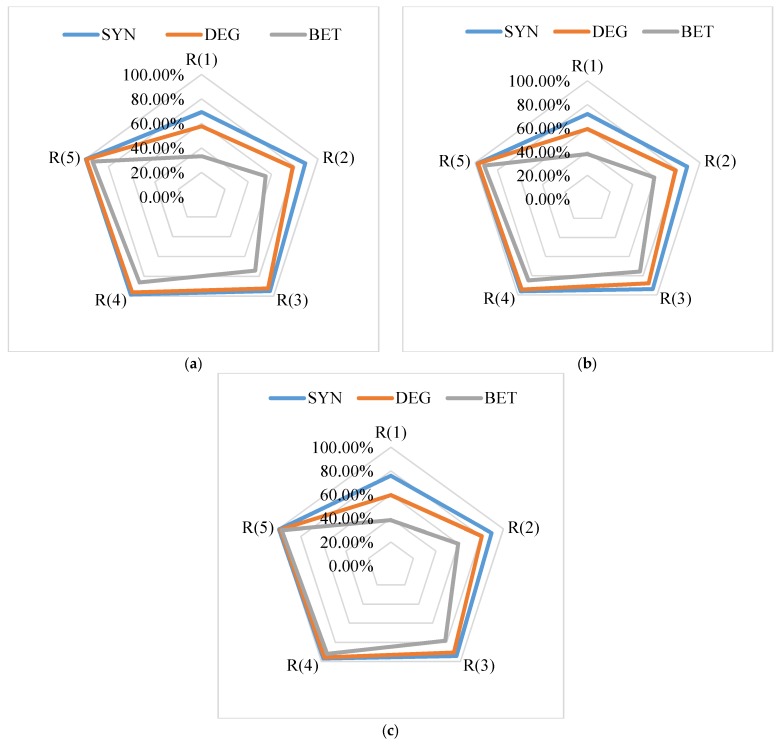
Average recognition rates of susceptible fusion gene in each interval when $N_{i}=15$: (**a**) $N_{f}=150$; (**b**) $N_{f}=200$; and (**c**) $N_{f}=250$.

Figure 4 shows the $N_{i}=15$. *p*(1) results of the SYN algorithm. The recognition rate was about 70%, whereas those for the same interval obtained by the other two algorithms were less than 60%. The *p*(2) value of the SYN algorithm was around 90%, which means approximately 90% of the susceptible fusion genes can be found using the SYN algorithm from its top 20% sorted results. As the range continuously increased, the *p*(*i*) value increased as well. The differences among algorithms continually decreased and the recognition rates of all algorithms gradually approached 100%.

**Figure 5:**
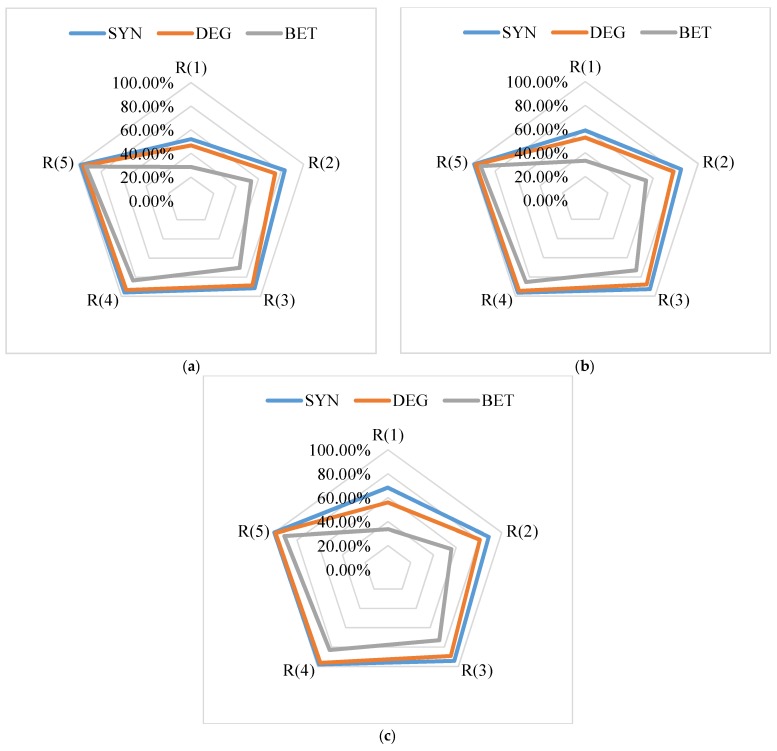
Average recognition rates of susceptible fusion gene in each interval when $N_{i}=25$: (**a**) $N_{f}=150$; (**b**) $N_{f}=200$; and (**c**) $N_{f}=250$.

Figure 5 highlights the statistical average results when $N_{i}=25$. Compared with the case when $N_{i}=15$, the overall recognition rate of the interval *R*(1) decreased significantly, which was mainly because the total number of fusion genes in *R*(1) was less than or equal to the number of pathogenic fusion genes in test samples ($N_{i}=25$). The corresponding proportion of pathogenic fusion genes was relatively lower. Other factors also affected the experimental results. We discuss the possible causes in the Discussion section. The *p*(2) value of the SYN algorithm was around 85%, whereas the recognition rates for the same interval obtained by the two other control algorithms were both less than 80%. The *p*(3) value of the SYN algorithm was higher than 90%, whereas the values obtained by the two control algorithms were less than 90%. Figure 4 and Figure 5 clearly illustrate that the recognition rate of the SYN algorithm in all situations was higher than those of the DEG and BET methods.

#### 2.2.3. Receiver Operating Characteristic Curve

**Figure 6:**
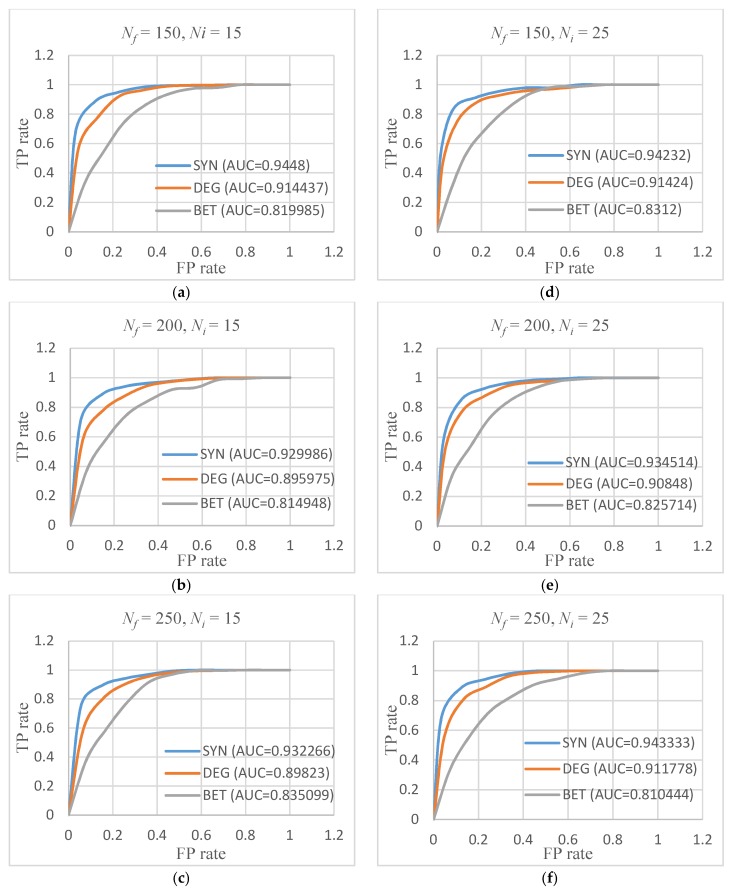
Receiver operating characteristic (ROC) curves of the three algorithms: (**a**) $N_{f}=150$, $N_{i}=15$; (**b**) $N_{f}=200$, $N_{i}=15$; (**c**) $N_{f}=250$, $N_{i}=15$; (**d**) $N_{f}=150$, $N_{i}=25$; (**e**) $N_{f}=200$, $N_{i}=25$; and (**f**) $N_{f}=250$, $N_{i}=25$. FP—false positive; TP—true positive.

By adding a classification boundary to the results of the algorithms, the original algorithm can be changed into a binary classification algorithm. Fusion genes above the classification limit can be classified as cancer pathogen fusion genes, and vice versa as normal fusion genes. As such, we calculated the algorithm’s receiver operating characteristic curve (ROC). Figure 6 shows the ROC curves of the three algorithms in all six cases and the area under curve (AUC) values for each curve, where the Y-axis is the true positive (TP) rate and the X-axis is the false positive (FP) rate.

From the ROC results, the best classification performance occurred at $N_{f}$=150 and $N_{i}=15$, where the AUC value was around 0.945. The situation with the smallest AUC score was $N_{f}=200$ and $N_{i}=15$, which had a value around 0.93. The overall performance of the proposed algorithm remained high.

## 3. Discussion

The experimental results clearly demonstrate that the proposed Synstable Fusion (SYN) algorithm performs better when calculating the importance of cancer-causing fusion genes in gene networks. More susceptible fusion genes were included in the top portion of the descending-sorted result, which means that possible oncogenic fusion genes have a greater tendency to be evaluated with higher importance values when using the SYN algorithm. As an example, three known oncogenic fusion genes found in the experimental results obtained by our algorithm received high importance rankings. The three fusion genes, EWSR1-FEV, HMGA2-LPP, and EWSR1-ETV4, were ranked first, tenth, and second in their respective datasets. Specific experimental datasets, importance calculation scores, and potential carcinogenic rankings are provided in Supplementary Table S2.

We found two phenomena worth noting. The first is that the number of cancer pathogenic fusion genes identified in the first 20% of the ranking results did not increase significantly as the total number of samples included in the test dataset increased. At $N_{i}=15$, the discrepancy among the maximum and minimum numbers of pathogenic fusion genes in the first 20% of the results of SYN algorithm was only 0.15 in three cases, and this difference only increased to 1.3 at $N_{i}=25$. The second phenomena is that the overall recognition effect slightly decreased when the total number of pathogenic fusion genes in the sample was high. For example, when $N_{f}=250$, the five-interval average recognition rate of the cancer-causing fusion gene of the SYN algorithm was 91.27%; when $N_{i}=15$, this value was 89.8%, and when $N_{i}=25$, a decrease of about 1.5% was observed. In the following, we discuss the possible causes of these two phenomena and explain why the results of the proposed algorithm are better than those of the other two algorithms.

The first case phenomenon occurred when the number of identified pathogenic fusion genes did not increase with the total fusion gene number in the sample. This may have occurred because the calculation scores of most disease-causing fusion genes were high but the scores of a fixed fraction of the proportion of susceptible fusion genes were lower. This is because the algorithms’ results are based on the genomics inference network derived by the classification algorithm of machine learning, and the result generated by classification algorithms must be partially consistent with the expected errors.

In the experimental dataset, the number of susceptible fusion genes was high whereas the recognition effect was slightly lower, possibly because the increase in the number of pathogenic fusion genes in the samples led to an increase in the occurrence probability of susceptible cancer fusion genes with low importance scores. The distribution of the number of pathogenic fusion genes in the first 20% results can provide support for this explanation. When $N_{i}=15$, the recognition distribution of each algorithm (Figure 2a) was almost unchanged, and the number of high-importance pathogenic fusion genes remained unchanged at a high rate. The number of identifications at $N_{i}=25$ (Figure 2b) slightly increased because some of the disease-causing fusion genes with slightly lower scores appeared in the test dataset. These genes were gradually identified as the range of recognition intervals increased. 

From the experimental results, the performance of the proposed algorithm is better than that of the DEG and BET algorithms under various parameter settings with experimental data. The proposed algorithm uses more comprehensive information contained in the gene network to calculate the importance of nodes. When evaluating the importance of nodes, BET algorithm only considers the influence of the nodes on the network topology, whereas the DEG algorithm only considers the influence between the node and its directly related parts of the network. However, in our algorithm, the “destructiveness equals to importance” hypothesis is applied, which not only considers the degree of the node, but also the impact of deleting the node on the network topology. This is equivalent to a certain degree of the incorporation of the first two algorithms. Therefore, SYN outperforms the DEG and BET algorithms.

## 4. Materials and Methods

The Synstable Fusion algorithm is based on the synchronous stability method, which evaluates the node importance according to the influence on stability of gene network when a node is removed. Wu et al. [36] used a Relevance Vector Machine (RVM)-based [48] ensemble-learning model to construct a whole gene network. This model integrates 17 heterogeneous genomic data and proteomics data [36]. We used this model mainly because it incorporates many different kinds of data, and simultaneously better handles the problem of missing attribute values among heterogeneous data [49] and outputs probabilistic results. Weighted edges existed between paired nodes in the entire gene network of the human genome. The weight represents the probability of interactive works between two genes, not only reflecting the direct interactions between genes, such as activation, inhibition, binding, and dissociation, but also other broader relationships among genes, for example, the likelihood of genes working on the same or similar biological pathways. In order to evaluate the influence of a node on stability, the synchronously stable networks were identified from the original gene network and the network of deleting a node, and then the difference between these two synchronously stable networks was calculated. Based on the node influence on network stability, the cancer fusion gene was evaluated. In this section, the design of the proposed algorithm is described in detail.

### 4.1. Synchronous Stability Method

In order to identify the synchronously stable network from a gene network, the synchronous stability method was required to ensure the relative stability of the gene network. A network is considered to be in synchronously stable state when it satisfies a certain condition.

#### 4.1.1. Synchronous Stability Condition

For the connected graph with ring of 2*K* adjacent nodes, the condition of synchronous stability is presented [35] as:

(1) $$w>\varepsilon =\frac{a}{n}{\left(\frac{n}{2K}\right)}^{3}\left(1+\frac{65}{4}\frac{K}{n}\right)$$

where w denotes the edge weight, ε represents the lowest limit of the *w*, *a* is an important parameter indicating the coupling state of network, *n* denotes the node amount of the network, and *K* indicates the number of half neighbors. Parameter *a* is called the coupling parameter that describes the coupling characteristic of the network. The value of *a* is determined by analyzing the adjacency matrix of graph. A previous study [50] indicated that $\lambda_{2}$ is the algebraic connectivity of the connected graph and $a<\lambda_{2}$. By inducing the Laplace matrix, we obtained a series of eigenvalues that satisfy $0=\lambda_{1}\leq \lambda_{2}\leq \ldots \leq \lambda_{n}$. The algebraic connectivity $\lambda_{2}$ is one of the eigenvalues and it was the minimum nonzero-eigenvalue. $\lambda_{2}$ denotes the synchronous ability of the connected graph. Thus, $0<a<\lambda_{2}$ and then we sequentially chose the fittest *a* value from this range based on some system analysis.

**Figure 7:**
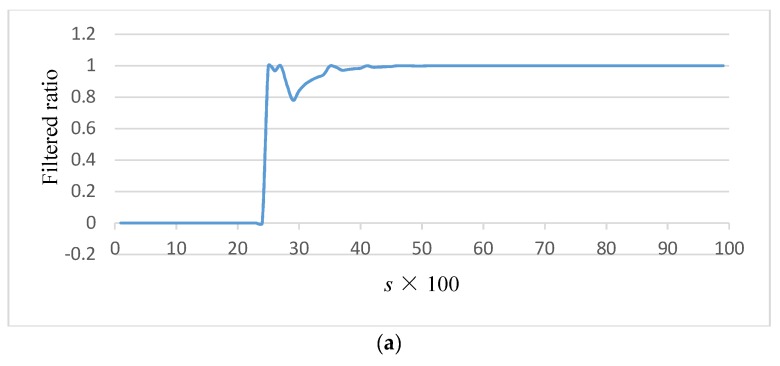

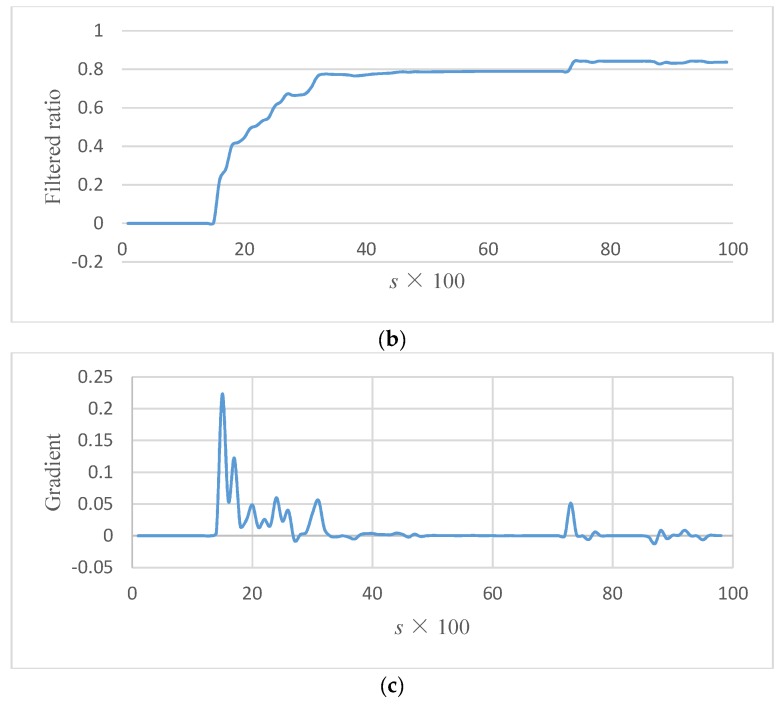
The results by various *s* (the product factor of coupling state parameter) values: (**a**) result of one set of data; (**b**) average result of 20 sets of data; and (**c**) Gradient of average result.

In order to find the most suitable value *a*, let $a=s\lambda_{2}$ and *s* = [0.01, 0.02, ..., 0.99]. For each *s* value, we calculated the proportion of the lost information filtered by the synchronously steady state of a given gene network. In the experimental gene data, 20 gene networks were randomly selected and generated. Figure 7 shows the result of one set of data, the average result of 20 sets of data, and the gradient of the average result.

We tried to find a suitable *s* value for most experimental data to filter out most noise and insignificant information while retaining key information in gene network. From Figure 6a,b, we found that depression points always occurred in the proportion of filtered data in all 20 experimental results. Through analyzing the gradient of the average data, we found the depression point where the gradient was first close to zero. From Figure 6c, point 0.28 satisfies the requirement. So $a=0.28\lambda_{2}$, which is inserted into Equation (1):

(2) $$w>\varepsilon =\frac{0.28\lambda_{2}}{n}{\left(\frac{n}{2K}\right)}^{3}\left(1+\frac{65}{4}\frac{K}{n}\right).$$

Our research considered two situations of the connected graphs: the gene networks have a fully connected topology, and the gene networks do not have a fully connected topology. The synchronous stability condition of fully connected networks can be obtained by letting *n =* 2*K.* Therefore, Equation (2) becomes:

(3) $$w>\varepsilon =2.555\frac{\lambda_{2}}{n}.$$

If the topology of graph is not fully connected, its maximum fully connected subgraph can be found. Let *m* denote half of the number of nodes in this subgraph. For the connected graph with a maximum ring of 2*K* adjacent nodes, the fully connected subgraph is a ring of 2*m* adjacent nodes, so we obtain 2*m <* 2*K*. The value of ε is increased by replacing *k* with *m*:

(4) $$w>\varepsilon =\frac{0.28\lambda_{2}}{n}{\left(\frac{n}{2m}\right)}^{3}\left(1+\frac{65}{4}\frac{m}{n}\right).$$

The edge weight limit ε calculated by Equation (4) is greater than the lowest limit of the synchronously stable condition. Therefore, the new limit can also be used as the judging condition for synchronous stability.

#### 4.1.2. Identification of Synchronously Stable Network

**Figure 8:**
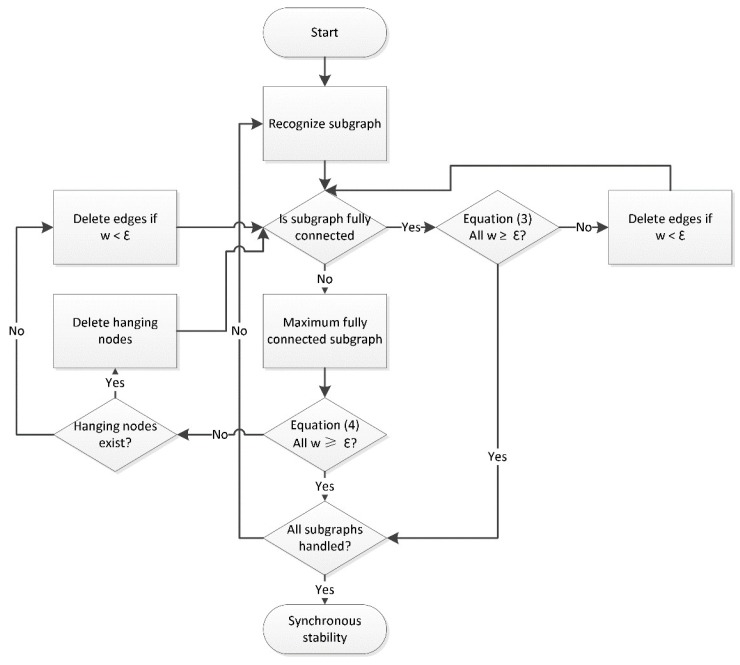
Flowchart followed for synchronously stable network identification.

From Equations (3) and (4), we obtained the lower edge weight limit for every gene network of various topologies. The network is in a synchronously steady state if $\forall w_{ij}\;$> ε, where $w_{ij}$ denotes the edge weight between node *i* and *j.* Otherwise, if $\exists w_{ij}\;$< ε, it is in a non-synchronously steady state and needs further processing to achieve synchronous stability. Different procedures were assigned based on whether or not the gene network was fully connected. All edges with a weight less than the lower limit were deleted if the network was fully connected. If the connected subgraph was a non-fully connected graph, the hanging nodes were detected. If there were hanging nodes, the hanging nodes were deleted. If no hanging nodes existed in the connected subgraph, then the edges with the weight less than the low limit were deleted. This procedure was iterated until all connected subgraphs were in the synchronously steady state. Figure 8 shows the flowchart of the identification of the synchronously stable network.

In the identification process for non-fully connected graphs, the maximum fully connected subgraph had to be obtained. This is called the maximum clique problem and is *NP*-hard that no known algorithms can achieve optimized solution. To solve this problem, some widely used algorithms include the greedy search algorithm, intelligent search algorithm, and heuristic search algorithm. In this work, we chose the greedy search algorithm.

### 4.2. Evaluation Susceptible Fusion Gene

Here, we describe the algorithm for prioritizing the susceptible cancer fusion gene using graph theory and gene network. First, we estimated the importance of gene nodes in the gene network. Then, the cancer susceptibility of the fusion genes was evaluated based on the importance of the partner gene nodes. The algorithm estimates the importance of gene nodes by evaluating the destructiveness to the network of deleting the node, where the destructiveness is evaluated according to the difference between the synchronously stable networks before and after the removal of the node. To ensure the gene network stayed synchronously stable, an identification method for synchronously stable networks was used. Figure 8 shows the process followed for achieving a synchronously stable network.

#### 4.2.1. Network Difference Evaluation

The impacts of deleting a node and its associated edges include two aspects: the impact on the degree of remained nodes, and the influence on connectivity. Considering these two aspects, we defined the metric for a gene network, *M*(*G*), as the ratio of total edge weights of network to the number of subgraphs:

(5) $$M\left(G\right)=\frac{\sum_{i\in G}\sum_{j\neq i\wedge j\in G}w_{ij}}{m_{G}}$$

where *G* indicates the gene network, $m_{G}$ denotes the number of subgraphs of *G*, and $w_{ij}$ denotes the weight between nodes *i* and *j*, i,j∈G. Once a node is deleted, the total edge weights decrease and the network connectivity decreases as well, which can be reflected by the increasing of number of subgraphs. All these influences can decrease the value of *M*(*G*). The network difference *D*(*G,v*) in deleting node *v* can be represented as:

(6) $$\begin{array}{ll} D\left(G,v\right) & =M\left(G\right)-M\left(G-v\right)\\ & =\frac{\sum_{i\in G}\sum_{j\neq i\wedge j\in G}w_{ij}}{m_{G}}-\frac{\sum_{i\in \left(G-v\right)}\sum_{j\neq i\wedge j\in \left(G-v\right)}w_{ij}}{m_{G-v}} \end{array}$$

where G−v is the network obtained by removing node v and its corresponding edges from network *G*. Based on the difference *D*(*G,v*), the importance *H*(*G,v*) of node *v* in network *G* is defined as:

(7) $$H\left(G,v\right)=\frac{D\left(v\right)}{M\left(G_{s}\right)}=\frac{M\left(G_{s}\right)-M\left({\left(G_{s}-v\right)}_{s}\right)}{M\left(G_{s}\right)}$$

where $G_{s}$ and ${\left(G_{s}-v\right)}_{s}$ represent the network *G* in synchronously stable state depending on whether or not node *v* is deleted.

#### 4.2.2. Calculation of Gene Node Importance

The algorithm for evaluating a gene node uses the synchronous stability method. Let *G =* (*V, W*) represent the gene network. *n* is the number of nodes in the network, $V=\left\{v_{1},v_{2},\ldots ,v_{n}\right\}$ indicates the set of nodes, and $W=\left\{w_{01},w_{02},\ldots ,w_{ij},\ldots ,w_{nk}\right\}$ represents the set of edge weights, where $w_{ij}$ is the edge weight between nodes *i* and *j*, i, j∈V. The algorithm processes are as follows:

Step 1: Utilize the susceptible cancer gene test data to generate gene network *G* from the human gene network.
Step 2: Process G by the synchronously stable network identification procedures described in Section 4.1.2., marked as $G_{s}$.
Step 3: Delete a node $v_{i}$ and its associated edges in $G_{s}$, $\left(G_{s}-v_{i}\right)$.
Step 4: Use the synchronously stable network identification to process $\left(G_{s}-v_{i}\right)$ to obtain the synchronously stable state, ${\left(G_{s}-v_{i}\right)}_{s}$.
Step 5: Use Equation (6) to calculate the D(G,v) value, and subsequently calculate the H(G,v) value using Equation (7).
Step 6: Evaluate the importance of every node in the gene network by repeating Steps 3–5.

#### 4.2.3. Evaluation of Susceptible Cancer Fusion Genes

By using the importance of the partner gene nodes, the significance of the fusion gene could be evaluated. The significance of a fusion gene is calculated by adding partner genes’ importance together then multiplying the weight of the edge between two partners. The significance *S*(*f*) of fusion gene *f* is:

(8) $$S\left(f\right)=\left(1+w_{ij}\right)\left(H\left(i\right)+H\left(j\right)\right)$$

where *i* and *j* denote the partner gene node associated with *f*, $w_{ij}$ is the edge weight between *i* and *j*, and *H*(*i*) and *H*(*j*) are the significance values of *i* and *j*, respectively. Fusion genes are formed by the interaction of partner genes. Edge weight between partner gene nodes reflects the interactive relationship between partners genes. Therefore, we consider this probabilistic value when evaluating the fusion gene’s significance.

## 5. Conclusions

This study proposed a method called Synstable Fusion for prioritizing the importance of fusion nodes in a weighted graph, based on the synchronous stability of gene network. This method, when applied to a gene network, effectively evaluates important fusion genes and identifies possible cancer pathogenicity fusion genes. The experimental results showed that the effectiveness of the proposed algorithm is superior to the other two algorithms based on network fusion centrality. In the experiment, we also found some issues that need attention, which could be the focus of future research and development. First, a more accurate gene network generation method should be explored to increase the reliability of the evaluation calculations. Second, other relevant theories can be applied instead of the synchronous stability method to achieve a more efficient and accurate interference information filtering method. In addition, we will try to introduce other algorithms that consider the node’s effect on network topology, so that we can more accurately evaluate the value of a node in the network.

## Supplementary Materials

The following are available online, Table S1: Lists of genes used in experiment, Table S2: Experimental results of datasets including known oncogenic fusion genes.
