# Supplementary material for: Synstable Fusion: A Network-Based Algorithm for Estimating Driver Genes in Fusion Structures
Source: Molecules. 2018 Aug 16;23(8):2055. doi: 10.3390/molecules23082055 (PMC6222865; doi:10.3390/molecules23082055)
Supplement: Supplementary file 1 [file molecules-23-02055-s001.zip › molecules-326990 supplementary for final/Supplementary for final.pdf]

Supplementary Description

S1: Supplementary Table S1 includes a list of 699 oncogenes from cancer gene census project and a list of 40230 genes from the whole-gene network. The oncogene list contains the HGNC gene symbols and corresponding NCBI Entrez Gene IDs. The network gene list only includes the NCBI Entrez Gene IDs.

S2: Supplementary Table S2 includes 3 tables of experimental datasets and results. Each dataset contains a professional validated oncogenic fusion gene (given a "c" mark in first column of table) among its susceptible fusion genes. Information columns included in tables are whether or not curated by expert, HGNC gene symbols and NCBI Entrez Gene IDs of partner genes of every susceptible fusion gene, NCBI Entrez Gene IDs of partner genes of every ordinary fusion gene, and the normalized importance value of each fusion gene. Table rows are descendingly sorted by the importance values.
